# An economic perspective on Malawi's medical "brain drain"

**DOI:** 10.1186/1744-8603-2-12

**Published:** 2006-12-18

**Authors:** Richard Record, Abdu Mohiddin

**Affiliations:** 1 Trade and Private Sector Development, Ministry of Industry, PO Box 30366, Capital City, Lilongwe 3, Malawi; 2 Division of Health and Social Care Research, Guy's, King's and St Thomas' School of Medicine, Kings College London, London SE1 3QD, UK

## Abstract

**Background:**

The medical "brain drain" has been described as rich countries "looting" doctors and nurses from developing countries undermining their health systems and public health. However this "brain-drain" might also be seen as a success in the training and "export" of health professionals and the benefits this provides. This paper illustrates the arguments and possible policy options by focusing on the situation in one of the poorest countries in the world, Malawi.

**Discussion:**

Many see this "brain drain" of medical staff as wrong with developed countries exploiting poorer ones. The effects are considerable with Malawi facing high vacancy rates in its public health system, and with migration threatening to outstrip training despite efforts to improve pay and conditions. This shortage of staff has made it more challenging for Malawi to deliver on its Essential Health Package and to absorb new international health funding.

Yet, without any policy effort Malawi has been able to demonstrate its global competitiveness in the training ("production") of skilled health professionals. Remittances from migration are a large and growing source of foreign exchange for poor countries and tend to go directly to households. Whilst the data for Malawi is limited, studies from other poor countries demonstrate the power of remittances in significantly reducing poverty.

Malawi can benefit from the export of health professionals provided there is a resolution of the situation whereby the state pays for training and the benefits are gained by the individual professional working abroad. Solutions include migrating staff paying back training costs, or rich host governments remitting part of a tax (e.g. income or national insurance) to the Malawi government. These schemes would allow Malawi to scale up training of health professionals for local needs and to work abroad.

**Summary:**

There is concern about the negative impacts of the medical "brain-drain". However a closer look at the evidence for and against the medical "brain-drain" in Malawi suggests that there are potential gains in managing medical migration to produce outcomes that are beneficial to individuals, households and the country. Finally we present several policy options.

## Background

With a GDP per capita of just USD 167 in 2004, Malawi remains one of the poorest countries in the world. Economic growth rates during the last 10–15 years have consistently fallen below that required to make an impact on poverty, and on most socio-economic indicators Malawi compares unfavourably with her regional neighbours. Malawi's exports are dominated by commodities (like tobacco) where the terms of trade are turning against the country. A number of efforts have been made by the Government of Malawi to move into new export sectors however the poor economic climate (high interest rates and inflation), the poor state of utilities and very high transport costs have hampered them.

Yet, in recent years Malawi has achieved notable success in the export of services – the export of skilled medical personnel – without any policy effort. Trade in services is increasingly being given more attention as a potential source of foreign exchange for land-locked countries such as Malawi that are struggling to compete in the world trading system and appear to have no comparative advantage in any product sector. But this "brain drain" has been described as rich countries "looting" doctors and nurses from developing countries [1]. The Malawi press has also described how the "nurses brain drain" is resulting in significantly reduced quality of care in public hospitals in the country [2,3]. The costs of the "brain-drain" are perceived as being much greater than the gains and include loss of public educational investment, intellectual capital, fewer and poorer health services, and understaffing of services [4-7].

Some recent research, however, has suggested that health sector migration might be a "win-win" for both developing and developed countries, if properly managed [8]. The purpose of this paper is to explore the costs and benefits of this migration to Malawi, and to determine what policy measures are required.

## Discussion

### The scale of the brain drain in Malawi and mitigation attempts

Many commentators in both the developed and developing world see this "brain drain" of essential medical staff from poor and HIV/AIDS afflicted countries such as Malawi as something which is fundamentally wrong. At first glance these arguments are compelling and draw upon the idea that developed countries are exploiting the developing world by taking the few trained medical staff away from essential work.

In a recent and full analysis, the Ministry of Health in Malawi has described the human resource situation as "critical" [9]. As can be seen in Table 1, the level of vacancies across the entire public health system is acute with an overall vacancy rate of 33 percent. However, this figure masks the severe shortage of nurses where 64 percent of established posts are unfilled. For surgeons and various types of doctor, the vacancy ratio reaches close to 100 percent. Of Malawi's 156 public sector doctors, 81 are working in central hospitals meaning that some districts do not have any doctor at all. Clinical Officer posts are much better filled (73%).

**Table 1:** Established posts and vacancies in Malawi's public health system, 2004.

Grade	No. of established posts	Filled
		
		%
Total (health professionals)	21,337	77
Nurses (all grades)	6,084	36
Clinical officers	3,852	73
Medical assistants	692	47
Surgeons	115	15
Medical specialists	65	5
Anaesthesiologists	14	29
Pathologists	22	0
Obstetricians/gynaecologists	126	9
Paediatricians	60	8

*Source*: Malawi Ministry of Health 2004 [9]

The MoH document continues to state that the average number of nurses in health centres is approximately 1.9, an indication that many are run with one or none at all and indeed, some health centres are now manned as health posts by Health Surveillance Assistants with as little as ten weeks of training.

In its national development plans, the Government of Malawi provides for the implementation of an Essential Health Package (EHP) of services costing USD 17 equivalent per person, yet the Ministry of Health itself estimates that the number of facilities with adequate staff able to implement this target is just 9.2 percent of the total number of facilities. The EHP staffing ratio of 2-2-1 (2 nurses, 2 medical assistants, and 1 clinical officer) per facility has now been revised downwards for internal monitoring purposes. This is a tacit admission on the part of government that Malawi's health human resources crisis is unlikely to be resolved in the near future.

Malawi's own figures for nurses and midwives leaving Malawi and seeking validation of certificates is running at just over 100 per year. In 2002, of the 103 who left, 83 went to the UK, with the remainder divided between the US, New Zealand, South Africa, Zimbabwe and Botswana. In 2003, 108 nurses and midwives left, with 90 going to the UK. Recent reports in June 2006 suggest that this migration pattern is continuing to outstrip Malawi's current annual training rate of around 60 nurses per year [10].

In reality, the number leaving Malawi may be even higher as some nurses emigrate to pursue careers other than nursing, in the care industry or otherwise and therefore not requiring qualification validation certificates, are not recorded in the statistics above.

The Project Appraisal Document of a USD 15 million World Bank Health Sector Support Project notes that:

*"The exodus of health workers out of [Malawi's] civil service started in early 2000 and was precipitated largely by the erosion of salaries, although there are other systemic underlying causes such as poor working conditions, and lack of drugs and medical supplies to work with" *[11].

Interestingly, the same document notes that in as recently as 1997/98 the level of vacancies for skilled staff was just 4.8 percent and there were no vacancies at all at the senior levels. Hence, the "medical brain drain" is a relatively new phenomenon as far as Malawi is concerned.

In October 2004, the Government of Malawi launched a major Sector-wide Approach (SWAp) for the health sector that attempted to revitalise Malawi's health services and support the delivery of the Essential Health Package. The SWAp programme of work saw the pooling of funds from major donors to the sector (UK, Norway and the World Bank) into the Ministry of Health budget to cover delivery of the EHP, strengthening of human resources, and systems strengthening and referral over a seven year period. The total cost of the SWAp is USD 735.7 million, of which 71 percent is to be provided by external donors [12]. The Government of Malawi also committed itself to raising the share of Government spending allocated to health from 11.2 percent in the 2002/03 budget to 13.5 percent by the end of the programme in 2009/10.

40 percent of the cost of the SWAp is allocated to strengthening human resources, of which a significant proportion is targeted towards raising the salaries of Malawi's public health workers. Table 2 shows the pre-October 2004 salaries for selected grades, and the post-October 2004 salaries which include changes in the official salary and a top-up funded using UK contributions to the SWAp.

**Table 2:** Salary structures for doctors and nurses in Malawi's public health system, 2005.

Grade		Currency*	Basic gross monthly salary (pre Oct-04)	Basic gross monthly salary (post Oct-04)	DFID top-up	Total gross monthly salary
Senior Physician	P4	MWK	29,153	126,000	66,040	192,040
		USD	243	1,050	550	1,600
Mid-level Physician	P5	MWK	25,216	34,853	20,100	54,953
		USD	210	290	168	458
Junior Physician	P8	MWK	22,100	23,297	14,246	37,543
		USD	184	194	119	313
Senior Nurse	PO/CTO	MWK	18,320	20,364	12,721	33,085
		USD	153	170	106	276
Mid-level Nurse	TO	MWK	12,930	13,849	8,917	22,766
		USD	108	115	74	190
Junior Nurse	TA	MWK	8,109	5,645	4,391	10,036
		USD	68	47	37	84

*Source: *MoH 2005 [13]

* based on average exchange rate during 2005 of USD 1= MWK 120

Senior physicians have seen the most dramatic increases in salaries and the gross P4 monthly salary has risen from USD 243 to USD 1,600. However, salaries at most grades have risen to the order of 40–60 percent. Mid-level nurse gross monthly salaries have risen from USD 108 to USD 190.

Yet the reality is that the remuneration gap for skilled medical staff between say, the UK and Malawi is so great, that these increases are likely to do little to reduce the incentives for staff to migrate. In the UK a newly qualified nurse earns £19,166 (USD 33, 290), a new junior physician £30,433 (USD 52,871), and a new senior physician £69,991 (USD 121,556) [14]. For newly qualified nurses, junior doctors and senior physicians this is still equivalent to around ten or more times the equivalent in Malawi. Hence it is hardly surprising that the exodus continues. However, to be fair the primary objective of the SWAp-funded salary top-up is not to compete with international labour markets, but to lift Malawi's health workers out of poverty, to ensure that workers receive at least a "satisfying level of income", and to discourage workers from leaving the health profession within Malawi.

### Causes of the brain-drain in Malawi and elsewhere

A major cause of the brain-drain in Malawi is the wage differential as described above. This is reinforced by a 2001 study of migration from sub-Saharan African countries by Hatton and Williamson that finds the two most important factors likely to fuel emigration are the real wage gaps between sending and receiving countries and, the demographic booms in the low-wage sending countries [15]. On the basis of their results, the authors find that the situation in the region is similar to the one in Europe in the late nineteenth century which fuelled mass migration.

Dovlo highlights a number of "push" and "pull" factors that contribute to migration among skilled health workers in Africa [16]. The major "push" factors include low remuneration, poor working conditions and low job satisfaction (particularly lack of equipment and medication which can significantly reduce job satisfaction). "Pull" factors include aging populations in developed countries and globalisation related market changes that reduce the transactions and search costs associated with medical migration.

Pond looked at the conditions in four developed countries and found that whilst the above arguments (ageing populations etc) have an influence, specific policy factors within these countries have equally caused an increase in demand for healthcare staff (and policies are amenable to change in a favourable way): for example, increases in the level of health spending, and the easing of entry regulations (both immigration and licensing) partly due to the time-lagged characteristics of the supply of health professionals (training takes at least six years for doctors, three years for nurses) [17]. A good example is the recent UK government's considerable increase in national health care funding and subsequent demand for staff (in the face of national shortages), hence the need to look for overseas sources that led to an easing of restrictions on migration.

Malawi's limited exploitable natural resources, combined with high population density and high poverty has meant that the country has a long history of migration. In the early post-independence years of the 1960s and 1970s, the major destination for official migration were the mines of South Africa. In 1972, for instance, remittances accounted for some 7 percent of GDP and 35 percent of total exports. However by the early 1990s, and for a number of reasons, the number of Malawians working in South Africa's mines had dwindled to almost none [18]. While the work in South Africa's mines was far from risk-free, these jobs were valued in Malawi due to the relatively high pay and official nature of the positions. Since the demise of such opportunities, Malawians have looked for other means to migrate. In 2000, it was estimated that some 17.4 percent of Malawi's skilled workforce was working abroad [19].

The demand for skilled health professionals by rich countries is likely to increase as their population's age and require more health (and social) care. Attempts to stem this "brain drain" have not been successful – the UK has initiated a policy of banning the active recruitment of healthcare staff from the poorest countries (the only country to do so), however this may not be working effectively as it faces practical challenges and infringes individuals' human rights. Indeed, there are more foreign-trained nurses on the UK nurse register than new British trained recruits [6,7].

### An alternative view: migration and remittances

The "brain drain" crisis can also be seen as a process that Malawi can theoretically capitalise on. Virtually all of Malawi's major export sectors are struggling to compete on world markets, yet without any policy effort whatsoever Malawi has demonstrated its competitiveness in the training (or "production") of doctors and nurses. Such exports allow Malawi to bypass the formal trade facilitation challenges (from the World Trade Organisation) that so hamper exports. The remittances from migrants are particularly important aspect of this alternative view. Unfortunately, data on remittances into Malawi is very limited and so it is not possible to measure the scale or impact of remittances from overseas workers, let alone the specific impact of remittances earned by skilled medical personnel working abroad. Therefore we present here international evidence as well.

### Remittances: the international evidence

Recent research has shown that international remittances are becoming one of the fastest growing and principle sources of foreign exchange for many least developed countries. Global remittances to developing countries reached USD 160 billion in 2004, considerably larger than ODA flows (USD 79 billion) and almost equal to foreign direct investment flows to developing countries (USD 166 billion) [20]. In fact, remittances are almost certainly underreported by perhaps up to 50 percent, implying that the returns to international migration are the dominant form of financial flows into developing countries.

In some countries remittances are a major source of services exports and foreign exchange. Mexico's annual remittance inflow has risen rapidly over recent years and now reaching USD 20 billion annually, is second only to petroleum as a generator of national wealth. Remittances also bring in more foreign exchange than tea exports in Sri Lanka, more than tourism in Morocco, and in Jordan, Lesotho, Nicaragua, Tonga and Tajikistan, they provide more than a quarter of gross national product [21].

Remittances also have a strong impact on poverty as they tend to go direct to poor households in developing countries, unlike official development assistance which is channelled through various development agencies and national governments and therefore has a much reduced pro-poor impact at the household level. World Bank analysis on Uganda, Bangladesh and Ghana has shown that the flow of international remittances have reduced poverty by 11, 6 and 5 percent respectively [20]. Work by Adams and Page has shown that an increase of 10 per cent in a country's share of international migrants leads to a 2 percent decline in poverty (measured in US$ per day terms) [22].

A recent survey of internationally recruited nurses working in London found that 57 percent of respondents regularly send money home, rising to over 60 percent for Africans [23]. 20 percent of the nurses were remitting more than a quarter of their monthly earnings.

Studies from other countries have shown that the remittances can have a number of positive impacts at the micro level beyond just supporting consumption among the poor. Work by Hanson and Woodruff shows that in Mexico, households with a migrant member complete significantly more years of schooling [24]. In Sri Lanka, De and Ratha find that remittance income has a significant positive impact on the weight of children under five years of age [25].

As with other capital constrained developing countries, the poor in Malawi are frequently limited in their access to finance for investment purposes. A study by Mesnard shows that during the 1980s, 87 percent of entrepreneurial projects started by Tunisian return migrants were fully financed by their own savings while abroad [26]. Yang finds that remittances have a major impact on reducing the credit constraints to new businesses in the Philippines [27].

In another paper, and using a household survey data from Western Kenya, Reardon finds that inflows of remittances from migrants are positively correlated with increased demand for education and construction activities in rural areas [28].

### Remittances: the Malawi evidence

Analysing data from the 1998 household living standards survey of Malawi, Chipeta and Kachaka note that while migrants' remittances are not the main source of income for poor Malawian households, they are significant [18]. In 1998, 20.3 percent of poor households received remittances and these remittances accounted for 4.9 percent of total per capita consumption and 6.3 percent of total per capita daily income. Migration of Malawians (and therefore the potential for remittances) has increased steadily since the 1998 household living standards survey, and in fact data from the 2005 round shows that "other current transfers" account for 9.5 percent of household income, rising to 14.2 percent of female-headed households [29]. Chipeta and Kachaka also argue that remittances to Malawi are counter-cyclical and therefore act as a pro-poor cushion during economic downturns [18].

Lucas studies the impact of remittances on Malawi, Botswana and Lesotho from workers employed in South African mines. The author finds that the short run decline in agricultural productivity due to the loss of labour is more than offset by later increases in productivity when remittances are utilised in farm investments [29].

### Type of migration

A major policy concern from countries that see large scale emigration, is whether or not migration is temporary or permanent. Migrants of either kind represent a loss of labour to the sending country, but if migration is only temporary then this loss might be seen as an investment in that when migrants eventually return, they are likely to bring back improved skills, expertise and knowledge ("brain gain"). Most commentators also tend to agree that temporary migrants remit a larger share of income than permanent migrants.

In contrast, permanent migration leads to a permanent loss of labour and for skills that demonstrate social spill-overs or effects (i.e. medical personnel), then the cost of departure invariably exceeds the cost of training. Where this cost is borne or subsidised by the state, then the effect is that sending countries (such as Malawi) are effectively investing in developed country public health.

Amin and Fruend argue that although the emigration of skilled workers directly reduces their number, a higher emigration rate might increase the stock of skilled workers in an economy by increasing the individual incentive to become trained in a particular skill which is in demand abroad [30]. The authors continue by noting that by increasing the expected returns to education, migration increases the demand for education, and thus potentially the eventual stock of educated workers.

Essentially this situation represents a "market failure" in that the benefits of migrating are reserved primarily for the individual and family members who benefit from remittances (although there is also an argument that remitted income consumed or invested is likely to have significant knock-on effects through the receiving economy), while the costs of the "brain drain" are borne by the state (through training costs) and the wider health consuming public (that pays for training through the tax system).

## Conclusions and policy recommendations

Migration has been, and is likely to continue to be a feature of Malawian society and economy with the medical "brain-drain" its latest incarnation. There is a case for seeing this as a success to be built upon rather than vilified. Strategies that aim to limit the movement of persons are likely to be ineffective at best – approaches instead need to optimise the impact of greater international mobility through a better understanding of the linkages between poverty, migration and development [31]. Taking a longer-term view and one that looks beyond an individual sector to the benefits to the whole country would suggest that migration and remittances have the potential to improve health and social outcomes. This means that the influence of donors (like the UK's DFID) which tends to focus on a specific sector and have a shorter timescale, should be secondary to those of the country as a whole.

In order for Malawi to fully capitalise and benefit from the export of skilled medical personnel the major challenge is to resolve the incidence of "market failure" whereby the costs of training medical staff lays with the state, but the benefits of working abroad are privately accrued. Essentially the uncosted effects of the presence of skilled health professionals in a country such as Malawi need to be costed and incorporated in the incentives framework of health workers that may or may not chose to emigrate.

One solution may be for the state to charge fees for medical training in Malawi that would be written-off pro rata over a given number of years of public service during which the doctor or nurse worked within Malawi. Staff would then be able to choose to emigrate and pay off the fees through overseas earnings, or to work off the debt through public service write-offs within Malawi.

Such a scheme would then allow Malawi to significantly scale up the training of medical personnel in order to train/produce for both the domestic health system/market and the for abroad/the export market. Goladfarb et al have explored this option with respect to deliberately training physicians "for export" in the Philippines [32].

Amin and Freund also make the point that that some sort of loan scheme might be an effective means of mitigating the loss of skilled medical personnel [30]. In the context of the ongoing Economic Partnership Agreement (EPA) negotiations between the countries of Eastern and Southern Africa and the EU, the authors recommend that an EPA, while providing for improved (so-called Mode 4) access to the EU, should also provide training and technical assistance to compensate African governments which carry the cost of training skilled workers, which then emigrate to the EU.

Temporary migration schemes have recently been advocated as a way of maximising the potential of migration by the UN Global Commission on International Migration [33,34]. A recent joint study by the COMESA Secretariat and the Commonwealth Secretariat proposes a "managed temporary migration scheme" for nurses, with a pilot programme for four sending countries, including Malawi [35]. The study includes a number of recommendations to "ensure" that migrant health professionals return to their country of origin. Some of the recommendations are valid, such as recognising experience earned abroad in the career progression structures in sending countries. However other recommendations, such as ensuring that work permits are not renewed after a specified period of time may violate the human rights of the migrant. In reality, any scheme which attempts to force migrants to return home through compulsion, rather than offering fair incentives, is likely to fail and cause undue stress. In addition, a managed scheme where migrants are selected through an official government programme, rather than based on merit-based recruitment in the market, is likely to be open to corruption and distortion.

Skeldon makes the entirely correct point that the medical brain drain is as much internal to an economy, as it is international [19]. Hence, efforts to restrict, "manage" migration, or make would-be migrants less attractive as professionals elsewhere, will not solve the problem of poor incentives for staff to remain in under-resourced public health systems, such as in Malawi. Similarly, salary top-ups such as under the Malawi Health SWAp, while attractive on one level, are unlikely to ever be of the magnitude needed to stem international departures from Malawi's public health system (although they may reduce the incentives for health personnel to move out of their profession, but remain in Malawi).

Another potential solution that has been presented is to separate medical professional training in developing countries into two tracks: an "advanced training programme" and a "basic training programme". Staff trained on the basic programme would be not qualified enough to be recruited abroad but would help address the some health needs of the population and are cheaper to train and employ – indeed Dovlo in a review finds that there can also be minimal differences in patient outcomes between clinical officers and doctors [36]. The Clinical Officer scheme in Malawi is doing this already and its expansion presents a policy option. Such officers also work in rural areas too, where doctors are scarcer. The scale-up of antiretroviral therapy is underway with such lower cadre health workers in Malawi as part of the 5-year antiretroviral therapy scale-up Plan (2006–2010) [37,38].

In terms of improving the incentives for migrants to remit, there is much that the Malawi Government could also do to facilitate increased remittances by migrants such as permitting the holding of foreign currency denominated accounts by Malawian's working abroad, without any requirement to convert foreign currency into Malawi kwacha. Reducing the cost of sending money from the UK to Malawi would also improve the incentives to remit, particularly for smaller, regular amounts. Remittances have risen significantly as wiring charges for sending money home have declined over the last ten years [39].

While it may also be tempting, from a social justice aspect to tax the remittances of migrant health workers, any policy measure that reduces the incentives to remit is likely to reduce the positive effects of remitting foreign currency earnings back to Malawi. A more workable solution might be for the Malawi Government to enter into an agreement with the UK Government (or wherever Malawian medical staff are working) whereby a portion of income tax or national insurance levied on migrant earnings in the UK is remitted to the Malawi Government for reinvestment in public health (highly cost-effective health interventions to guide policy to meet the health Millennium Development Goals are available [40]. Such a scheme would not affect the incentives of the migrant to remit as the income transfer would be purely from developed country government to developing country government.

In sum there are several policy options that could be used in the Malawi context as we have described above. The choice of particular options should be evidence based, and interdisciplinary in nature when assessing the relative costs and benefits of health personnel migration, and in matching where those relative costs and benefits accrue. Only by ensuring that when a Malawian health worker chooses to migrate, that decision making process take full account of the personal and societal costs of emigration, will the issue of "medical brain drain" be effectively, and fairly resolved.
